# Shedding light on trophic interactions: A field experiment on the effect of human population between latitudes on herbivory and predation patterns

**DOI:** 10.1002/ece3.10449

**Published:** 2023-08-31

**Authors:** Inés María Alonso‐Crespo, Juan Antonio Hernández‐Agüero

**Affiliations:** ^1^ Institute of Ecology Leuphana Universität Lüneburg Germany; ^2^ Senckenberg Gesellschaft für Naturforschung Frankfurt (am Main) Germany; ^3^ Department of Environmental Geography Vrije Universiteit Amsterdam Amsterdam The Netherlands

**Keywords:** bird predation, insect herbivory, latitude, UHI effect and urbanization

## Abstract

Interactions between species within an ecosystem (e.g. predation and herbivory) play a vital role in sustaining the ecosystem functionality, which includes aspects like pest control and nutrient cycling. Unfortunately, human activities are progressively disrupting these trophic relationships, thereby contributing to the ongoing biodiversity decline. Additionally, certain human activities like urbanization may further impact the intensity of these trophic interactions, which are already known to be influenced by latitudinal gradients. The aim of this study was to test the hypothesis of whether the impact of human population, used as a proxy for human pressure, differs between latitudes. To test it, we selected 18 study sites at two latitudes (i.e. ~53°N and ~50°N) with varying human population density (HPD). We used artificial caterpillars placed on European beech branches to assess bird predation and took standardized pictures of the leaves to estimate insect herbivory. Remote sensing techniques were used to estimate human pressure. We found that the intensity of bird predation varied in response to HPD, with opposite trends observed depending on the latitude. At our upper latitude, bird predation increased with HPD, while the opposite was observed at the lower latitude. Herbivory was not affected by urbanization and we found higher levels of herbivory in the lower compared to the higher latitude. At the lower latitude, certain species may experience a disadvantage attributed to the urban heat island effect due to their sensitivity to temperature fluctuations. Conversely, at the higher latitude, where minimum temperatures can be a limitation, certain species may benefit from milder winters. Overall, this study highlights the complex and dynamic nature of trophic relationships in the face of human‐driven changes to ecosystems. It also emphasizes the importance of considering both human pressure and latitudinal gradients when assessing the ecological consequences of future climate change scenarios, especially in urban environments.

## Graphical abstract

This study tests how human pressure affects trophic interactions at different latitudes, being the first to demonstrate experimentally the opposite effects of human presence on trophic interactions between latitudes.

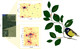

## Resumen

Las interacciones entre especies dentro de un ecosistema (p. ej., depredación y herbivoría) juegan un papel vital en el mantenimiento de la funcionalidad del ecosistema, incluyendo aspectos como el control de plagas y el ciclo de nutrientes. Desafortunadamente, las actividades humanas están interrumpiendo progresivamente estas relaciones tróficas, contribuyendo así a la continua disminución de la biodiversidad. Además, ciertas actividades humanas, como la urbanización, pueden afectar aún más la intensidad de estas interacciones tróficas, que ya se sabe que están influenciadas por gradientes latitudinales. El objetivo de este estudio fue probar la hipótesis de si el impacto de la población humana, utilizada como indicador de la presión humana, difiere entre latitudes. Para probarlo, seleccionamos 18 sitios de estudio en dos latitudes (es decir, ~53°N y ~50°N) con densidad de población humana (HPD) variable. Utilizamos orugas artificiales colocadas en ramas de haya común para evaluar la depredación de aves y tomamos fotografías estandarizadas de las hojas para estimar la herbivoría de los insectos. Se utilizaron técnicas de teledetección para estimar la presión humana. Descubrimos que la intensidad de la depredación de las aves varió en respuesta al HPD, observándose tendencias opuestas según la latitud. En nuestra latitud superior, la depredación de aves aumentó con HPD, mientras que se observó lo contrario en la latitud inferior. La herbivoría no se vio afectada por la urbanización y encontramos niveles más altos de herbivoría en la latitud inferior en comparación con la superior. En latitudes bajas, ciertas especies pueden experimentar una desventaja atribuida al efecto de isla de calor urbano debido a su sensibilidad a las fluctuaciones de temperatura. Por el contrario, en latitudes más altas, donde las temperaturas mínimas pueden ser una limitación, ciertas especies pueden beneficiarse de inviernos más suaves. En general, este estudio destaca la naturaleza compleja y dinámica de las relaciones tróficas frente a los cambios en los ecosistemas provocados por el hombre. También enfatiza la importancia de considerar tanto la presión humana como los gradientes latitudinales al evaluar las consecuencias ecológicas de los futuros escenarios de cambio climático, especialmente en entornos urbanos.

## 1 INTRODUCTION

Nowadays, 54% of the world's human population lives in cities (≥300,000 inhabitants), and this percentage is expected to increase to 70% by 2050 (United Nations, 2018). Cities are highly disturbed areas that have recurrent problems of air, water pollution (Bai et al., 2017) and elevation of temperatures (Kim, 1992). They experience profound changes in land use, with fatal consequences for ecosystems, biogeochemical cycles and climate (Bai et al., 2017). Among the human activities that cause habitat loss, urban development has been identified as one of the primary contributors to high local extinction rates (McKinney, 2002). Urban areas have been shown to have adverse effects on various aspects of organisms, including their abundance, diversity and organismal properties such as size, reproduction and performance. Additionally, these human‐induced changes in urban environments can significantly impact species interactions like trophic interactions. Trophic interactions, as bird predation and insect herbivory, are particularly relevant for biological communities. They are responsible for important ecosystem services, such as nutrient cycling (DeAngelis, 2012), pest control (Whelan et al., 2008) and adaptation to the effects of climate change or human health (Sirakaya et al., 2018), which is especially important in cities.

The effects of urban human disturbance on trophic interactions have been extensively studied. Some studies have found a decrease in bird predation in more urbanized areas (Ferrante et al., 2014; Gering & Blair, 1999). Conversely, other studies have found the opposite (Cupitra‐Rodríguez et al., 2023; Jokimäki & Huhta, 2000; Thorington & Bowman, 2003). A recent meta‐analysis showed that reported bird predation in urban areas was significantly lower compared with rural areas (Eötvös et al., 2018), explained by the reduction of predators abundance produced by habitat loss, although some species could benefit from urbanization, such as generalist species. In line with this, the higher bird predation in big cities compared to rural areas found in Kozlov et al. (2017) was explained by an increase in resource availability in urban areas, which finally produced a trophic cascade effect resulting in lower herbivory rates in urban areas (top–down control). Besides, other authors have found a decrease in herbivory with the increase in human population either with a decrease in chewer herbivory (Meineke et al., 2019), chewer, leaf miner and galler herbivory (Valdés‐Correcher et al., 2022) or for chewer herbivory but not in other guilds (Moreira et al., 2019; Nuckols & Connor, 1995). Contrarily, other studies found an increase in herbivory abundance (Dale & Frank, 2014; Parsons & Frank, 2019) or intensity in chewer herbivory in urban areas (Christie & Hochuli, 2005; Cuevas‐Reyes et al., 2013; Rivkin & de Andrade, 2023). Abiotic changes in urban areas (e.g. urban heat island effect or water availability) can produce a reduction in the abundance of native species, although this effect appear to be species‐specific and could vary regionally, some species can acclimate to abiotic urban changes, many others cannot (Miles et al., 2019).

The influence of latitude on trophic interactions has been also widely studied, but no consensus has been reached on the matter. Different results have been observed, both across trophic levels and varying with latitude. Higher levels of invertebrate (Gray et al., 2022) and bird (Zvereva et al., 2019) predation have been documented at higher latitudes. However, invertebrate predation has also been found to increase at lower latitudes (Roslin et al., 2017) and decrease with increasing latitudes (Zvereva et al., 2019). For invertebrates (Lövei & Ferrante, 2017), birds and mammals (Roslin et al., 2017), no significant differences in predation intensity have been detected based on latitude. Furthermore, Romero et al. (2018) proposed that alterations in temperature might better explain the increased predation observed at lower latitudes rather than latitude alone. The effects of latitude on herbivory have been investigated as well. On one hand, some studies found that the herbivorous abundance (Pennings et al., 2009) and leaf herbivory (Adams & Zhang, 2009: Garibaldi et al., 2011; Kozlov, 2008; Kozlov et al., 2015; Moreira et al., 2015) increase towards the equator. On the other hand, several studies have not detected effects of the latitude over the herbivory pressure (Andrew & Hughes, 2005; del‐Val & Armesto, 2010; Kozlov, 2008; Moles et al., 2011; Moles & Westoby, 2003; Salazar & Marquis, 2012; Sinclair & Hughes, 2010).

Biotic interactions can be affected by latitudinal temperature changes (Frenne et al., 2013) as well as by other temperature changes, like the island heat effect caused by urbanization (Kim, 1992; Youngsteadt et al., 2015). Cities can be up to 10°C warmer than the surrounding rural areas, and this effect can be even greater at higher latitudes (Wienert & Kuttler, 2005). This feature makes cities an ideal place for exploring the ecological consequences of possible future scenarios of climatic change (Youngsteadt et al., 2015), as they currently experience temperatures that will be reached at the same latitudes in the future.

The investigation of how urbanization effects vary across latitudes remains relatively understudied. Kozlov et al. (2017) discovered that urban areas exhibited lower foliage mortality and higher levels of predation by ants and bird attacks over dummy preys in urban areas, irrespective of latitude. Similarly, Moreira et al. (2019) found lower foliage mortality caused by leaf chewers in urban areas compared to rural areas, and again this effect was not influenced by latitude. In a systematic review conducted by Hernández‐Agüero et al. (2023), the combined influence of latitude and urban factor was used as explanatory variables in order to understand changes in the intensity of herbivory and bird predation. The findings revealed a pattern where trophic interactions decreased with an increase in human population density at lower latitudes, no significant effect at intermediate latitudes, and an increase in trophic interactions with increasing urbanization at higher latitudes. In higher latitudes, winter temperatures act as a limiting factor for the survival of certain species. The intensification of urbanization and the consequent increase in mean temperatures at these latitudes may enhance survival and trophic relationships, while less climatic constraint at lower latitudes may remove this advantage, resulting in reduced survival and trophic interactions.

The objective of this study was to test whether the patterns detected in Hernández‐Agüero et al. (2023), what we called the ‘opposite latitudinal‐disturbance’ hypothesis, can be detected in a field experiment. The specific aims of this research were (i) to investigate the effects of human population density on trophic interactions (bird predation and insect herbivory) and (ii) to compare how these effects change with latitude. To this end, we studied herbivory and predation across a human population gradient at two latitudes in Germany. As proposed by Gering and Blair (1999), the gradient approach to studying the effect of urbanization allows researchers to evaluate the effects of an ecological gradient in the same way as temperature or moisture, rather than simply comparing categorical variables (urbanized—rural). We hypothesized fewer trophic interactions in response to human population density at lower latitude, and the opposite in higher latitude.

## 2 MATERIALS AND METHODS

### 2.1 Study sites

**FIGURE 1:**
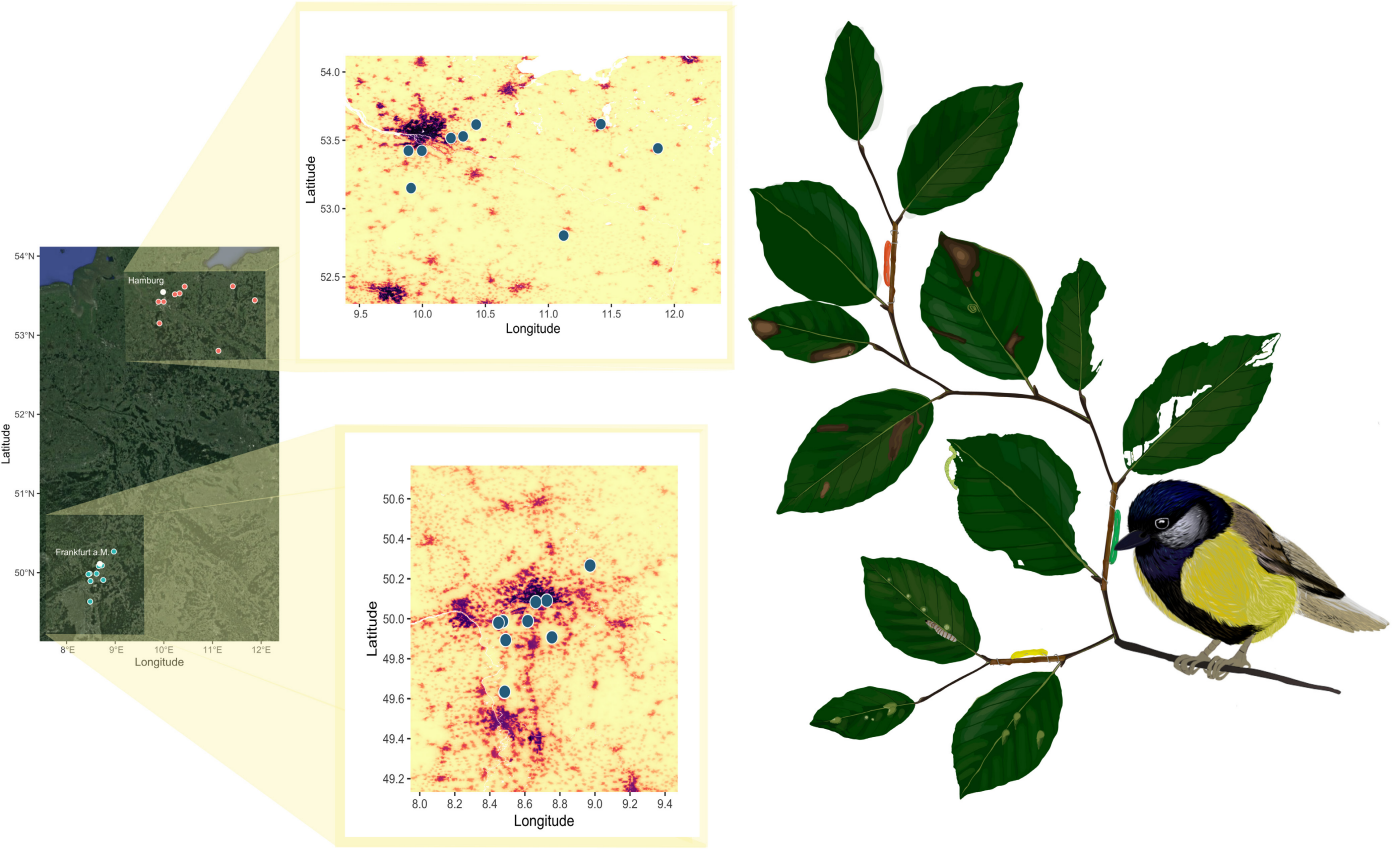
Location of study sites and graphical representation of trophic interaction studied. The yellow‐red scale represents the human population density.

In the summer of 2022, from May to July, 18 study sites at two different latitudes were selected [nine in northern Germany (53.39 ± 0.26°N) and nine in southern Germany (49.98 ± 0.17°N)] (Figure 1) to conduct a field experiment in areas with a range of human population density (HPD) between 42 and 2772 humans per km^2^ and to study the effect of urbanization on trophic interactions between latitudes. Germany was selected to answer this question because the human population is well spread throughout the country and is not clustered only in a few cities, so more replicates without spatial autocorrelation problems could be taken into account. The study sites were selected from 600.000 random points in Germany. These points were generated with the ‘seq’ function from the ‘base’ R package (R Core Team, 2021). From these, we selected only coordinates inside ‘green urban areas’, ‘broad‐leaved forest’ or ‘transitional woodland‐shrub’ categories from Corine Land Cover maps (CLC, 2018; Version 20), using the function ‘gIntersects’ from ‘rgeos’ package (Bivand & Rundel, 2021) ending with 3197 possible study sites. HPD was estimated for every random point with the ‘extract’ function from ‘raster’ package (Hijmans et al., 2020) after creating a 1.2 km buffer with ‘st_buffer’ function from ‘sf’ package (Pebesma & Bivand, 2018) using a high resolution (~100 × 100 m) layer from WorldPop Global Project Population Data (https://www.worldpop.org/). The layer was extracted for German boundaries using the function ‘Export.image.toDrive’ in Google Earth Engine (Gorelick et al., 2017). 1.2 km was selected to include enough environmental effects without overlapping with other study sites (minimal distance between sites ~2.5 km), but considering the resolution of the layer (~100 m). The latitudinal and population ranges obtained were divided into 10 categories and only one site per category of latitude and population density was selected. After this process, 67 different co‐ordinates were obtained, covering a broad range of latitudes and human population densities of Germany. Among them, in order to study the effect of latitude on trophic interactions, two latitudes (±0.5°) were selected (50.1° for the southern region and 53.3° for the northern one). These regions were chosen to avoid the potential effect of elevation from mountainous areas of southern Germany (Bayern) and to have a latitudinal difference between sites (3.2°). Because trophic interactions can be affected by elevation (Roslin et al., 2017), we selected sites with elevations lower than 200 m.a.s.l. This selection process resulted in 18 suitable study sites (nine in northern Germany and nine in southern Germany) that accomplished all the premises established for site selection. The HPD in the 1.2 km radius around our study sites ranged from 10 to 1535 people per km^2^ for the northern region (mean 565; SD: 607), and 151–2451 people per km^2^ in the southern region (mean: 905; SD: 846). Other studies (e.g. Valdés‐Correcher et al., 2022) showed that trophic interaction can be affected by the amount of built percentages or the proportion of vegetated areas. We obtained the percentage of built percentage for our study sites with a 1.2 km radius from Dynamic World V1 ‘built’ band at 30‐m resolution with the mode values and the NDVI median values from Landsat 8 30‐m resolution both between 01 January 2021 and 31 December 2021.

### 2.2 Species selection

Before the study commenced, the selected forests were visited to detect common species across them. *Fagus sylvatica* L. (European beech) was selected as the study species. *F. sylvatica* is a large deciduous tree whose distribution covers a high latitudinal gradient (27° from 37°N to 60°N; Durrant et al., 2016) and it is frequently present in German forest masses in the two selected latitudes.

### 2.3 Experimental design

#### 2.3.1 Predation

To determine whether birds' intensity of predation on caterpillars differs between latitudes and HPD, artificial caterpillars were placed in *F. sylvatica* trees in all the study sites. The caterpillars were made out of brown, green and yellow plasticine (Staedtler 8421; Mars Deutschland GmbH) to capture potential variations in colour prey preferences across different geographical locations and predator identities (Zvereva et al., 2019). Only one tree species was selected to place the plasticine caterpillars since predation can differ depending on the tree species (Hernández‐Agüero et al., 2020). Artificial caterpillar methodology has been widely used to investigate the bird predation with successful results (Allen et al., 1998; Howe et al., 2009; Remmel & Tammaru, 2009). The artificial caterpillars were made out of odourless, non‐toxic plasticine with a shape of 30 mm in length and 4 mm in diameter, and were attached to branches of trees with a 0.5‐mm wire (Rayher 24079000; Rayher Hobby GmbH) threaded longitudinally through each model. Five *F. sylvatica* trees were randomly selected from each site, but always at least 10 m apart from each other, and three caterpillars (one of each color) were placed on each one, with 270 caterpillars in all the experiment. Caterpillars were located on thin branches (3–10 mm) at a height of 1.5–2 m, and at least 30 cm apart from each other, which has been considered statistically independent in previous studies (Bereczki et al., 2014; Dáttilo et al., 2016; Tvardikova & Novotny, 2012). Caterpillars were left in the field for a period of 3 months starting on 9 May 2022, coinciding with the months of largest periods of light and bird activity. The caterpillars were checked every 4 weeks to assess the number of attacks on them, resulting in a total of three reviews per site. When any damage was found, it was counted as predated (1), and the caterpillars were reshaped to avoid duplication of damage counts between reviews. If a caterpillar was not found or was highly depredated, it was also counted as predated (1) and it was replaced with a new one. When the caterpillar did not show any sign of predation, it was counted as non‐predated (0). If the caterpillar and the wire were not found (e.g. cutted branches) it was excluded from the analysis (NA). The attack marks left on the plasticine caterpillars allowed us to identify bird or insect predation (Low et al., 2014). After three visits, the caterpillars were removed, including the wires. A total of 273 plasticine caterpillars from three colours were placed in 91 *F. sylvatica* trees during 77.28 days (SD: 4.25). Bird predation was estimated three times over the duration of the experiment, each 25.69 days (SD: 3.74).

#### 2.3.2 Herbivory

Insect herbivory was measured in the five *F. sylvatica* trees selected per site to study bird predation. From 1 July to 7 July, 20 leaves per tree were randomly selected and were photographed using a consistent methodology. All branches from which the leaves were selected could be reachable from the ground (at a height of 1–2 m). A total of 1821 *F. sylvatica* leaves were photographed in situ, using a folder with a transparent cover that allowed the introduction of the leaf and its flattening to take a proper scaled picture to analyse defoliation, mines and galls presence. Photographs were taken in situ without ripping out the leaves. This methodology was selected to avoid damage to the plant and to produce plant anti‐herbivory responses. Herbivory data were analysed by using the software ImageJ (Rueden et al., 2017). The images were digitally scaled using the known real scale present within the image itself. This scaling allowed us to accurately determine the total leaf, defoliated and mined area captured in the images. Visible leaf area was annotated as defoliation. Manual predictions of the whole leaf area were annotated as the total leaf area. Mines area was annotated as mines, and the number of galls per leaf was also annotated.

#### 2.3.3 Biodiversity characterization

Since species composition could affect biotic interactions (Muiruri et al., 2016), censuses of tree species were performed at each location. At each site, a measuring tape of 20 m was extended in the north direction from two randomly selected trees out of the five present. This approach was implemented to prevent sampling biases, and in this case, trees number 2 and 4 were randomly chosen before doing the census. The same methodology was consistently applied across all sites. The tape was walked from both sides with a measuring ruler of 1.5 m, including in the census all trees inside this path. Two sectors of 20 × 3 m (120 m^2^ in total) were sampled per study area. Leaves of unidentified species were collected for subsequent identification. All bird species observed or heard during visits to the study sites were annotated.

To reduce the environmental impact of the study by reducing the fuel consumption, the shorter path to visit driving all study sites selected by car was calculated with the ‘gmapsdistance’ package (Azuero‐Melo et al., 2018).

### 2.4 Data analysis

We used generalized linear mixed models (GLMMs) with a binomial, beta or Poisson error distribution and a logit link function to investigate the effects of the human population and latitude on bird predation and insect herbivory. Models with binomial error distribution were implemented for estimating the presence or absence of damage in plasticine caterpillars. Models with Poisson error distribution were implemented to estimate the effect of HPD and latitude in the number of galls detected in the leaves. Models with beta error distribution were implemented for estimating the effect of HPD and latitude in percentage of defoliation and mines. These models were developed with the function ‘glmmTMB’ from the ‘glmmTMB’ package (Brooks et al., 2017). Predicted variables were transformed following the suggestions of the authors as: (y*(*n* − 1) + 0.5)/*n*.

In the GLMMs for predation, we included the following random factors: (i) Larvae_ID and (ii) days after the last review/placement to account for possible predation probability due to the position of the larvae and the different intervals between reviews. In the GLMMs for herbivory, we included Tree_ID as a random factor to avoid pseudoreplication. HPD and built percentage, and HPD and NDVI values had correlation values of .58 and −.66, respectively; therefore, it was not possible to include those predictors in the model using HPD without violating model assumptions, and all the models were repeated for the two predictors. All models were also replicated for insect predation.

For both herbivory and predation alternative models were compared using the Akaike information criterion (AIC) to select the explanatory variables (i.e. fixed effects) and random effects. Models with a difference in AIC < 2 could not be reliably differentiated from the top model and thus model averaging occurs. Following Nakagawa and Schielzeth (2013), we estimated the *R* ^2^ of all plausible linear or mixed models. This allowed two components of *R* ^2^ to be calculated: (1) a marginal *R* ^2^ ($R_{m}^{2}$) that only considers the variability explained by fixed effects and (2) a conditional *R* ^2^ ($R_{c}^{2}$) that accounts for the variability supported by both fixed and random effects. Model residuals were explored using a simulation‐based approach to create readily interpretable‐scaled (quantile) residuals for the fitted GLMMs with the function ‘simulateResiduals’ from the ‘DHARMa’ package (Hartig, 2021). In addition, non‐parametric multivariate analysis of variance test made with the ‘adonis2’ function of the ‘vegan’ package (Oksanen et al., 2022) was used to test if both plant and bird species composition differs between sites.

The data frames used in these analyses were organized with tidyverse R package (Wickham et al., 2019). Plots were created using the R packages ‘ggplot2’ (Wickham, 2016), ‘ggpubr’ (Kassambara, 2020), ‘ggmap’ package (Kahle & Wickham, 2013) and the colour‐blind palette viridis from ‘Rvision’ package (Garnier et al., 2021). All the analyses were performed using the R environment (ver. 4.1.0; R Core Team, 2021).

### 2.5 Ethics

No specific permits were necessary to carry out this field experiment considering § 44 Abs. 1, Nr. 1 (BNatSchG) was not unfulfilled. All ‘Landkreis’ and ‘Bundesland’ were notified previously deploying the experimental design.

## 3 RESULTS

### 3.1 Predation

At the end of the experiment, 594 caterpillars were recorded as predated out of 819 total placed caterpillars. 75% of the caterpillars were predated, 23% non‐predated and 2% of the caterpillars were excluded from the analysis.

**FIGURE 2:**
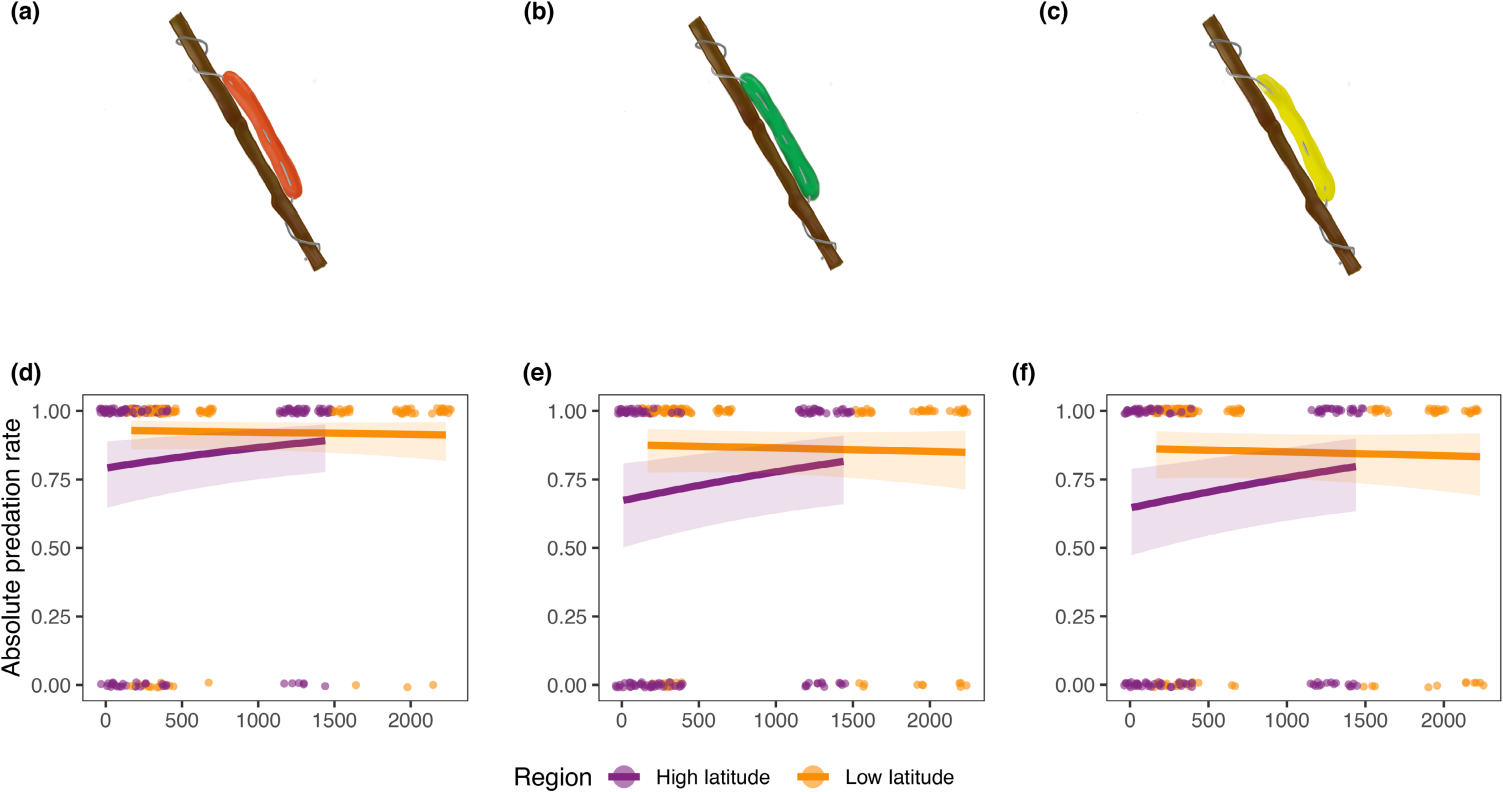
Predictions of generalized linear mixed models showing the absolute predation rate with 95% confidence intervals along a gradient of the human population density at the two regions studied for every colour. Each drawing represents the colour of the larvae to which the data in the graph below refers being (a, d) brown, (b, e) green and (c, f) yellow. Observed values of absolute predation are shown by dots.

The best explanatory model (Table 1) for bird predation included the colour of the caterpillar, latitudinal region, HPD and the interaction between HPD and latitudinal region. The impact of HPD on bird predation varied depending on the region. In the higher latitude, bird predation increased with higher HPD, while in the lower latitude bird predation decreased with higher HPD (Figure 2). Brown caterpillars were more predated than green (*p*‐value = .0251) or yellow (*p*‐value = .0051) caterpillars in all the sites. Effect sizes of the best model tables can be found in Table S1. No significant effects were observed on any of the variables (latitudinal region, colour and HPD) utilized to explain insect predation (Table 1).

**TABLE 1:** Comparison of alternative models for predation using the Akaike information criterion (AIC).

Predictors	Bird predation			Insect predation	
	df	AIC	$R_{m}^{2}/R_{c}^{2}$	df	AIC
HPD*Region*Colour	14	848.76		8	637.07
HPD*Region + Colour	**8**	**843.58**	**0.082/0.206**	8	637.07
HPD + Region + Colour	7	846.34		7	635.75
HPD + Colour	6	858.03		6	635.74
Region + Colour	6	845.79		6	633.91
Colour	5	860.06		5	633.75
Null model	3	866.78		**3**	**630.34**

*Note*: The best model (lowest AIC) is indicated in boldface type.

Abbreviations: $R_{c}^{2}$, conditional *R* ^2^ that accounts for the variability supported by both fixed and random effects; $R_{m}^{2}$, a marginal *R* ^2^ that only considers the variability explained by fixed effects.

### 3.2 Herbivory

**FIGURE 3:**
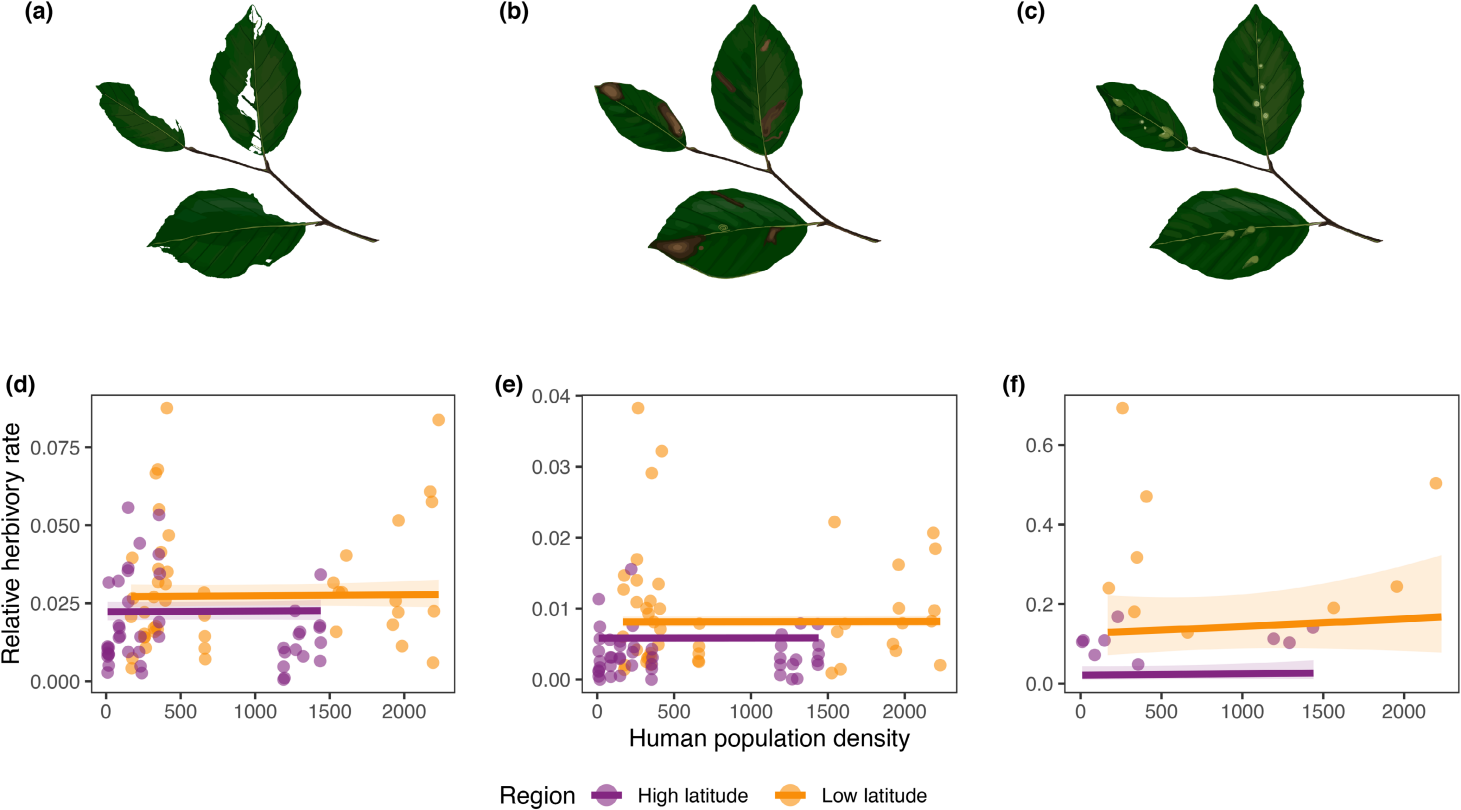
Predictions of generalized linear mixed models showing the 95% confidence intervals along a gradient of the human population density at the two regions studied for every type of herbivory studied. Each drawing represents the type of herbivory to which the data in the graph below refers being (a, d) defoliation, (b, e) mines and (c, f) galls. Observed values of herbivory are shown by dots.

Of the analysed leaves, 1215 were defoliated, 842 had mines and 190 presented galls. Mean percentage of defoliation was 2.38% (95% CI: 2.09%–2.67%), mean percentage of mines was 0.65% (95% CI: 0.56%–0.75%) and mean number of galls was 0.29 (95% CI: 0.24–0.35). The mean total leaf area estimated was 36.86 cm^2^ (95% CI: 36.16–37.55 cm^2^). For defoliated and mined area and the number of galls, we obtained two best explanatory models (Table 2). In one the latitudinal region was included and the other included the latitudinal region and the HPD as well. In the higher latitude defoliated area, mined area and the number of galls were smaller than in the lower latitude (Figure 3). Although one of the best models include HPD, the effect of this variable on herbivory was not significant. Effect sizes of the averaged best model tables can be found in Tables S2–S4. Best models for built surface and NDVI were equivalent to the best models of HPD (Tables S5–S8).

**TABLE 2:** Comparison of alternative models for herbivory using the Akaike information criterion (AIC).

Predictors	Chewer herbivory			Miner herbivory			Galler herbivory		
	df	AIC	$R_{m}^{2}/R_{c}^{2}$	df	AIC	$R_{m}^{2}/R_{c}^{2}$	df	AIC	$R_{m}^{2}/R_{c}^{2}$
HPD*Region	6	−12,138.41		6	−16,508.98		5	2265.27	
HPD + Region	**5**	**−12,139.29**	**0.008/0.081**	**5**	**−16,510.98**	**0.021/0.029**	**4**	**2263.75**	**0.151/0.563**
HPD	4	−12,135.99		4	−16,477.62		3	2278.77	
Region	**4**	**−12,140.92**	**0.008/0.081**	**4**	**−16,512.88**	**0.021/0.029**	**3**	**2263.16**	**0.145/0.566**
Null model	3	−12,136.61		3	−16,476.86		2	2281.49	

*Note*: The best model (lowest AIC) is indicated in boldface type.

Abbreviations: $R_{c}^{2}$, conditional *R* ^2^ that accounts for the variability supported by both fixed and random effects; $R_{m}^{2}$, a marginal *R* ^2^ that only considers the variability explained by fixed effects.

A total of 372 trees and shrubs were identified in the vegetation census (Table S9). Most common tree species were *Fagus sylvatica* (*n* = 123), *Acer platanoides* (*n* = 96) and *Carpinus betulus* (*n* = 37). The tree species composition did not differ between study sites (*R* ^2^ = .0761, *p*‐value > .05; Figure S1). Fourteen different bird species were detected at the study sites, with *Fringillia coelebs*, *Parus major* and *Turdus merula* being the most common bird species detected (Table S10). The bird species composition did not differ between study sites (*R* ^2^ = .1416, *p*‐value > .05; Figure S2).

The minimum distance to visit all study sites by car was calculated at 1419.64 km. The study sites were visited five times with a total of 1043 kg of CO_2_ emitted during the development of this experiment.

## 4 DISCUSSION

Our hypothesis, the ‘opposite latitudinal‐disturbance’, proposes that biotic interactions differ between high and low latitudes and these patterns can be diminished or enhanced by the HPD. Our data support this hypothesis partially (for bird predation). When bird predation was evaluated, we found that it varied with the HPD differently depending on the latitude.

In the higher latitude of study, bird predation increased with increasing HPD. These results have been evidenced in previous studies, which found the same pattern for bird predation (Cupitra‐Rodríguez et al., 2023; Jokimäki & Huhta, 2000; Kozlov et al., 2017; Thorington & Bowman, 2003). It is known that in urban environments there is a dominance of certain predators (the so‐called ‘urban exploiters’ in contrast to ‘urban avoiders’; sensu Blair, 1996), more adapted to these environments (Sorace, 2002). The presence of those ‘urban exploiters’ added to the exotic predators (Sasvári et al., 1995), conduct to an increase in predation pressure. At high latitudes, low temperatures limit the performance and survival of species (Currie et al., 2004; Deutsch et al., 2008). Urban areas produce local climate change that results in an increase in environmental temperatures (the urban heat island effect). This can directly increase species fitness and abundance by affecting their metabolism, development and fecundity (Bale, 2002; Dale & Frank, 2017).

In the lower latitude of study, bird predation decreased with increasing HPD. Previous studies have found the same results for birds (Eötvös et al., 2018; Gering & Blair, 1999) and invertebrates (Ferrante et al., 2014), the predatory pressure decreased in more urbanized areas. Increasing urbanization at lower latitudes could have a negative effect on species diversity because of habitat loss or fragmentation or the dominance of some species over the rest in the communities, especially predator species (Gray, 1989). All these factors decreased the number of niches available and the number of different species present in the ecosystems, finally reducing the predation pressure.

For insect predation, no effects were found for any variable. Neither latitude nor HPD affected insect predation. Opposite findings were detected in Ferrante et al. (2014), where insect predation was lessened from rural towards urban areas. In terms of latitude effect, higher levels of insect predation have been reported at lower latitudes (Lövei & Ferrante, 2017; Roslin et al., 2017; Zvereva et al., 2019). We did not find any of these effects with any of the variables of study or their interaction. The main explanation for the absence of evidence on insect predation, and one of the limitations of this study is that we installed the caterpillars to principally test bird predation that was our target trophic interaction jointly with insect herbivory. We only collected data of invertebrates that are able to reach the branches of the trees, missing many guilds with our methodology. In order to test invertebrate predation, we should have installed caterpillars at the ground level as well. Another potential explanation, which also serves as one of the primary limitations of this study, is the restricted inclusion of a latitudinal gradient. This decision was made in order to minimize the environmental variability present between latitudes.

Latitude affected herbivory. In the higher latitude, herbivory pressure was smaller than in the lower latitude. Regardless of the latitude, an increase in HPD was not associated with different levels of herbivory across any type of herbivory which disagree with other studies in herbivore abundance (Dale & Frank, 2017; Meineke et al., 2013; Parsons & Frank, 2019) or leaf defoliation (Cuevas‐Reyes et al., 2013; Rivkin & de Andrade, 2023). We explain our absence of effects of HPD on herbivory with the counter effects of biotic and abiotic factors occurring in urban areas. The fact that urban areas support fewer natural enemies because of a less complex vegetation (Keane & Crawley, 2002) has been used to explain the increase in herbivory with population density, due to the lack or reduction of biological control of pests (Dale & Frank, 2017; Meineke et al., 2014). This would result in less mortality of prey (Noske, 1998) and therefore an increase in herbivory. In addition, herbivore species could proliferate in urban areas because of a reduction in plant defence investment due to stress (White, 1969), due to an increase in plant growth caused by the fertilizing effect of nutrients and CO_2_ in urban areas (Price, 1991), or, especially ectotherm species, due to urban temperature increases (Youngsteadt et al., 2015). Meanwhile, abiotic factors such as water availability, pollution and fragmentation can reduce herbivorous species abundance (Miles et al., 2019).

Despite the small latitudinal gradient used in this study, we found differences in herbivory between latitudes, with lower levels of insect herbivory at the higher latitude. We also found lower levels of bird predation at higher latitude, but only for low HPD areas. More productive ecosystems (Dobzhansky, 1950; Novotny et al., 2006), explained by lower seasonality (Coley & Aide, 1991), sustain higher levels of herbivory (McNaughton et al., 1989). More diverse predator communities create redundancies and complementation in prey consumption that finally drives an increase in predation (Paine, 1966). Those processes could explain the results we have obtained for herbivory and, only in low populated areas, for predation. Several studies have also found that biotic interactions become more intense at lower latitudes (Schemske et al., 2009; Zvereva & Kozlov, 2021) on leaf defoliation (Adams & Zhang, 2009; Garibaldi et al., 2011; Kozlov, 2008; Kozlov et al., 2015; Moreira et al., 2015; Pennings et al., 2009), insect predation (Roslin et al., 2017; Zvereva et al., 2019) and bird predation (Matysioková & Remeš, 2022; Romero et al., 2018). Although, some studies found higher levels of seed predation (Moreira et al., 2015) or bird predation (Adams & Zhang, 2009; Zvereva et al., 2019) at higher latitudes. The authors attribute those results to the higher availability of natural prey, which may help explain the findings regarding bird predation in high population‐dense sites. This explanation aligns with our own results, as we observed slight increases in herbivory with higher HPD.

Only two studies have previously explored the interaction between HPD and latitude studying species diversity (Perez et al., 2022) or biotic interactions (Hernández‐Agüero et al., 2023). Opposite effects of latitude and urbanization have been recently detected on ant species diversity in a 60° absolute latitudinal range study across 63 cities (Perez et al., 2022). At lower latitudes, cities were relatively species‐poor and harboured distinct ant communities relative to nearby non‐urban communities. In higher‐latitude cities, both species richness and community composition were more similar to the surrounding non‐urban ant communities. Regarding biotic interactions, the intensity of herbivory and predation in a global analysis decreased with an increase in HPD at lower latitudes, remained unaffected at intermediate latitudes, and increased at higher latitudes (Hernández‐Agüero et al., 2023). Cities are ideal places to explore the ecological consequences of possible future climatic change scenarios (Youngsteadt et al., 2015). For predation, predictions of global patterns due to climate change, adding the increase of temperature and the increase of climatic instability will result in a general decrease in predation pressure over time, which could alter the functioning of terrestrial ecosystems and their associated ecosystem services (Romero et al., 2018). Nevertheless, we only detected a coincidence of these two assumptions for our lower latitude, possibly representing the unexpected effects of urbanization at our higher latitude in response to climate change.

## 5 CONCLUSIONS

Throughout this study, the ‘opposite latitudinal‐disturbance’ has been confirmed for bird predation. Bird predation rates increase with increasing HPD at higher latitude and bird predation rates decrease with increasing human population density at lower latitude. This shows synergic effects between both variables on higher trophic interactions. This could imply important effects on ecosystem services (e.g. nutrient cycling, pest control) that would differently affect different regions, producing a disruption on the natural balance of ecosystems (i.e. ecological imbalance). Further investigation is needed to determine to what extent these effects could be detected in other trophic levels, at more distant latitudes with more ample human population density range.

## AUTHOR CONTRIBUTIONS

**Inés María Alonso‐Crespo:** Data curation (equal); formal analysis (equal); investigation (equal); methodology (equal); visualization (lead); writing – original draft (equal); writing – review and editing (equal). **Juan Antonio Hernández‐Agüero:** Conceptualization (lead); data curation (equal); formal analysis (equal); funding acquisition (lead); investigation (equal); methodology (equal); project administration (equal); software (lead); visualization (supporting); writing – original draft (equal); writing – review and editing (equal).

## FUNDING INFORMATION

This study was supported by *Sociedad Española de Etología y Ecología Evolutiva* grants ‘Convocatorias de Ayudas a la Investigación de la SEEEE Año 2022’.

## CONFLICT OF INTEREST STATEMENT

The authors confirm to not have any conflict of interest.

### OPEN RESEARCH BADGES

This article has earned Open Data, Open Materials and Preregistered Research Design badges. Data, materials and the preregistered design and analysis plan are available at [https://doi.org/10.6084/m9.figshare.22670152.v1].

## Supporting information


Appendix S1.

## Data Availability

All data and R script are available at figshare (https://doi.org/10.6084/m9.figshare.22670152.v1).

## References

[ece310449-bib-0001] Adams, J. M. , & Zhang, Y. (2009). Is there more insect folivory in warmer temperate climates? A latitudinal comparison of insect folivory in eastern North America. The Journal of Ecology, 97(5), 933–940. 10.1111/j.1365-2745.2009.01523.x

[ece310449-bib-0002] Allen, J. A. , Raison, H. E. , & Weale, M. E. (1998). The influence of density on frequency–dependent selection by wild birds feeding on artificial prey. Proceedings of the Royal Society of London. Series B: Biological Sciences, 265(1400), 1031–1035. 10.1098/rspb.1998.0395

[ece310449-bib-0003] Andrew, N. R. , & Hughes, L. (2005). Herbivore damage along a latitudinal gradient: Relative impacts of different feeding guilds. Oikos, 108(1), 176–182. 10.1111/j.0030-1299.2005.13457.x

[ece310449-bib-0004] Azuero‐Melo, R. , Rodriguez, D. , & Zarruk, D. (2018). Gmapsdistance: Distance and travel time between two points from Google Maps . R package version 3.4. https://CRAN.R‐project.org/package=gmapsdistance

[ece310449-bib-0005] Bai, X. , McPhearson, T. , Cleugh, H. , Nagendra, H. , Tong, X. , Zhu, T. , & Zhu, Y.‐G. (2017). Linking urbanization and the environment: Conceptual and empirical advances. Annual Review of Environment and Resources, 42(1), 215–240. 10.1146/annurev-environ-102016-061128

[ece310449-bib-0006] Bale, J. S. (2002). Insects and low temperatures: From molecular biology to distributions and abundance. Philosophical Transactions of the Royal Society of London. Series B, Biological Sciences, 357(1423), 849–862. 10.1098/rstb.2002.1074 12171648 PMC1693004

[ece310449-bib-0007] Bereczki, K. , Ódor, P. , Csóka, G. , Mag, Z. , & Báldi, A. (2014). Effects of forest heterogeneity on the efficiency of caterpillar control service provided by birds in temperate oak forests. Forest Ecology and Management, 327, 96–105. 10.1016/j.foreco.2014.05.001

[ece310449-bib-0008] Bivand, R. , & Rundel, C. (2021). Rgeos: Interface to geometry engine – Open source (‘GEOS’) . R package version 0.5‐9. https://CRAN.R‐project.org/package=rgeos

[ece310449-bib-0009] Blair, R. B. (1996). Land use and avian species diversity along an urban gradient. Ecological Applications, 6(2), 506–519. 10.2307/2269387

[ece310449-bib-0010] Brooks, M. E. , Kristensen, K. , van Benthem, K. J. , Magnusson, A. , Berg, C. W. , Nielsen, A. , Skaug, A. J. , Maechler, M. , & Bolker, B. M. (2017). glmmTMB balances speed and flexibility among packages for zero‐inflated generalized linear mixed modeling. The R Journal, 9(2), 378–400. 10.32614/RJ-2017-066

[ece310449-bib-0011] Christie, F. J. , & Hochuli, D. F. (2005). Are declines in tree health more than an edge effect? Ecology and Society, 10(1), 10.

[ece310449-bib-0012] Coley, P. D. , & Aide, T. M. (1991). Comparison of herbivory and plant defenses in temperate and tropical broad‐leaved forests. In P. W. Price , T. M. Lewinsohn , G. W. Fernandes , & W. W. Benson (Eds.), Plant‐animal interactions: Evolutionary ecology in tropical and temperate regions (pp. 5–49). John Wiley.

[ece310449-bib-0013] Corine Land Cover (CLC) . (2018). *Micka SIEUSOIL Hub: Corine Land Cover (CLC)* 2018, version 20. https://hub.sieusoil.eu/record/full/5e139af5‐6348‐4a7b‐9060‐17480a000073

[ece310449-bib-0015] Cuevas‐Reyes, P. , Gilberti, L. , González‐Rodríguez, A. , & Fernandes, G. W. (2013). Patterns of herbivory and fluctuating asymmetry in *Solanum lycocarpum* St. Hill (Solanaceae) along an urban gradient in Brazil. Ecological Indicators, 24, 557–561. 10.1016/j.ecolind.2012.08.011

[ece310449-bib-0016] Cupitra‐Rodríguez, J. , Cruz‐Bernate, L. , & Montoya‐Lerma, J. (2023). Attack rates on artificial caterpillars in urban areas are higher than in suburban areas in Colombia. Journal of Tropical Ecology, 39, e19. 10.1017/S026646742300007X

[ece310449-bib-0017] Currie, D. J. , Mittelbach, G. G. , Cornell, H. V. , Field, R. , Guegan, J.‐F. , Hawkins, B. A. , Kaufman, D. M. , Kerr, J. T. , Oberdorff, T. , O'Brien, E. , & Turner, J. R. G. (2004). Predictions and tests of climate‐based hypotheses of broad‐scale variation in taxonomic richness. Ecology Letters, 7(12), 1121–1134. 10.1111/j.1461-0248.2004.00671.x

[ece310449-bib-0018] Dale, A. G. , & Frank, S. D. (2014). The effects of urban warming on herbivore abundance and street tree condition. PLoS One, 9(7), e102996. 10.1371/journal.pone.0102996 25054326 PMC4108386

[ece310449-bib-0019] Dale, A. G. , & Frank, S. D. (2017). Warming and drought combine to increase pest insect fitness on urban trees. PLoS One, 12(3), e0173844. 10.1371/journal.pone.0173844 28278206 PMC5344462

[ece310449-bib-0020] Dáttilo, W. , Aguirre, A. , De la Torre, P. L. , Kaminski, L. A. , García‐Chávez, J. , & Rico‐Gray, V. (2016). Trait‐mediated indirect interactions of ant shape on the attack of caterpillars and fruits. Biology Letters, 12(8), 20160401. 10.1098/rsbl.2016.0401 27484648 PMC5014034

[ece310449-bib-0021] DeAngelis, D. L. (2012). Dynamics of nutrient cycling and food webs (Vol. 9). Springer Science & Business Media.

[ece310449-bib-0022] del‐Val, E. , & Armesto, J. J. (2010). Seedling mortality and herbivory damage in subtropical and temperate populations: Testing the hypothesis of higher herbivore pressure toward the tropics. Biotropica, 42(2), 174–179. 10.1111/j.1744-7429.2009.00554.x

[ece310449-bib-0023] Deutsch, C. A. , Tewksbury, J. J. , Huey, R. B. , Sheldon, K. S. , Ghalambor, C. K. , Haak, D. C. , & Martin, P. R. (2008). Impacts of climate warming on terrestrial ectotherms across latitude. Proceedings of the National Academy of Sciences of the United States of America, 105(18), 6668–6672. 10.1073/pnas.0709472105 18458348 PMC2373333

[ece310449-bib-0024] Dobzhansky, T. (1950). Evolution in the tropics. American Scientist, 38(2), 209–221.

[ece310449-bib-0025] Durrant, T. H. , De Rigo, D. , & Caudullo, G. (2016). *Fagus sylvatica* in Europe: Distribution, habitat, usage and threats. In European atlas of forest tree species. European Commission. https://w3id.org/mtv/FISE‐Comm/v01/e012b90

[ece310449-bib-0026] Eötvös, C. B. , Magura, T. , & Lövei, G. L. (2018). A meta‐analysis indicates reduced predation pressure with increasing urbanization. Landscape and Urban Planning, 180, 54–59. 10.1016/j.landurbplan.2018.08.010

[ece310449-bib-0027] Ferrante, M. , Lo Cacciato, A. , & Lövei, G. L. (2014). Quantifying predation pressure along an urbanisation gradient in Denmark using artificial caterpillars. European Journal of Entomology, 111(5), 649–654. 10.14411/eje.2014.082

[ece310449-bib-0028] Frenne, P. , Graae, B. J. , Rodríguez‐Sánchez, F. , Kolb, A. , Chabrerie, O. , Decocq, G. , Kort, H. , Schrijver, A. , Diekmann, M. , Eriksson, O. , Gruwez, R. , Hermy, M. , Lenoir, J. , Plue, J. , Coomes, D. A. , & Verheyen, K. (2013). Latitudinal gradients as natural laboratories to infer species' responses to temperature. The Journal of Ecology, 101(3), 784–795. 10.1111/1365-2745.12074

[ece310449-bib-0029] Garibaldi, L. A. , Kitzberger, T. , & Ruggiero, A. (2011). Latitudinal decrease in folivory within *Nothofagus pumilio* forests: Dual effect of climate on insect density and leaf traits? Global Ecology and Biogeography, 20(4), 609–619. 10.1111/j.1466-8238.2010.00623.x

[ece310449-bib-0030] Garnier, S. , Ross, N. , Rudis, R. , Camargo, A. P. , Sciaini, M. , & Scherer, C. (2021). Revision – Colorblind‐friendly color maps for R . R package version 0.6.2.

[ece310449-bib-0031] Gering, J. C. , & Blair, R. B. (1999). Predation on artificial bird nests along an urban gradient: Predatory risk or relaxation in urban environments? Ecography, 22(5), 532–541.

[ece310449-bib-0032] Gorelick, N. , Hancher, M. , Dixon, M. , Ilyushchenko, S. , Thau, D. , & Moore, R. (2017). Google earth engine: Planetary‐scale geospatial analysis for everyone. Remote Sensing of Environment, 202, 18–27. 10.1016/j.rse.2017.06.031

[ece310449-bib-0033] Gray, H. L. , Farias, J. R. , Venzon, M. , Torres, J. B. , Souza, L. M. , Aita, R. C. , & Andow, D. A. (2022). Predation on sentinel prey increases with increasing latitude in brassica‐dominated agroecosystems. Ecology and Evolution, 12(7), e9086. 10.1002/ece3.9086 35845383 PMC9272068

[ece310449-bib-0034] Gray, J. S. (1989). Effects of environmental stress on species rich assemblages. Biological Journal of the Linnean Society, 37(1–2), 19–32. 10.1111/j.1095-8312.1989.tb02003.x

[ece310449-bib-0035] Hartig, F. (2021). DHARMa: Residual diagnostics for hierarchical (multi‐level/mixed) regression models . R package version 0.4.4. https://CRAN.R‐project.org/package=DHARMa

[ece310449-bib-0036] Hernández‐Agüero, J. A. , Polo, V. , García, M. , Simón, D. , Ruiz‐Tapiador, I. , & Cayuela, L. (2020). Effects of prey colour on bird predation: An experiment in Mediterranean woodlands. Animal Behaviour, 170, 89–97. 10.1016/j.anbehav.2020.10.017

[ece310449-bib-0037] Hernández‐Agüero, J. A. , Ruiz‐Tapiador, I. , Garibaldi, L. A. , Kozlov, M. V. , Mäntylä, E. , Nacif, M. E. , Salinas, N. , & Cayuela, L. (2023). The effects of human population density on trophic interactions are contingent upon latitude. bioxriv [preprint]. 10.1101/2023.08.22.554272

[ece310449-bib-0038] Hijmans, R. J. , van Etten, J. , Sumner, M. , Cheng, J. , & Beva, A. (2020). Raster: Geographic data analysis and modeling . R package version 3.6‐3. https://CRAN.R‐project.org/package=raster

[ece310449-bib-0039] Howe, A. , Lövei, G. L. , & Nachman, G. (2009). Dummy caterpillars as a simple method to assess predation rates on invertebrates in a tropical agroecosystem. Entomologia Experimentalis et Applicata, 131(3), 325–329. 10.1111/j.1570-7458.2009.00860.x

[ece310449-bib-0040] Jokimäki, J. , & Huhta, E. (2000). Artificial nest predation and abundance of birds along an urban gradient. The Condor, 102(4), 838–847. 10.1093/condor/102.4.838

[ece310449-bib-0041] Kahle, D. , & Wickham, H. (2013). Ggmap: Spatial visualization with ggplot2. The R Journal, 5(1), 144–161. 10.32614/RJ-2013-014

[ece310449-bib-0042] Kassambara, A. (2020). Ggpubr: ‘ggplot2’ based publication ready plots . R package version 0.4.0. https://CRAN.R‐project.org/package=ggpubr

[ece310449-bib-0043] Keane, R. M. , & Crawley, M. J. (2002). Exotic plant invasions and the enemy release hypothesis. Trends in Ecology & Evolution, 17(4), 164–170. 10.1016/S0169-5347(02)02499-0

[ece310449-bib-0044] Kim, H. H. (1992). Urban heat Island. International Journal of Remote Sensing, 13(12), 2319–2336. 10.1080/01431169208904271

[ece310449-bib-0045] Kozlov, M. V. (2008). Losses of birch foliage due to insect herbivory along geographical gradients in Europe: A climate‐driven pattern? Climatic Change, 87, 107–117. 10.1007/s10584-007-9348-y

[ece310449-bib-0046] Kozlov, M. V. , Lanta, V. , Zverev, V. , Rainio, K. , Kunavin, M. A. , & Zvereva, E. L. (2017). Decreased losses of woody plant foliage to insects in large urban areas are explained by bird predation. Global Change Biology, 23(10), 4354–4364. 10.1111/gcb.13692 28317226

[ece310449-bib-0047] Kozlov, M. V. , Stekolshchikov, A. V. , Söderman, G. , Labina, E. S. , Zverev, V. , & Zvereva, E. L. (2015). Sap‐feeding insects on forest trees along latitudinal gradients in northern Europe: A climate‐driven patterns. Global Change Biology, 21(1), 106–116. 10.1111/gcb.12682 25044643

[ece310449-bib-0048] Lövei, G. L. , & Ferrante, M. (2017). A review of the sentinel prey method as a way of quantifying invertebrate predation under field conditions. Insect Science, 24(4), 528–542. 10.1111/1744-7917.12405 27686246

[ece310449-bib-0049] Low, P. A. , Sam, K. , McArthur, C. , Posa, M. R. C. , & Hochuli, D. F. (2014). Determining predator identity from attack marks left in model caterpillars: Guidelines for best practice. Entomologia Experimentalis et Applicata, 152(2), 120–126. 10.1111/eea.12207

[ece310449-bib-0050] Matysioková, B. , & Remeš, V. (2022). Stronger negative species interactions in the tropics supported by a global analysis of nest predation in songbirds. Journal of Biogeography, 49(3), 511–522. 10.1111/jbi.14321

[ece310449-bib-0051] McKinney, M. L. (2002). Urbanization, biodiversity, and conservation: The impacts of urbanization on native species are poorly studied, but educating a highly urbanized human population about these impacts can greatly improve species conservation in all ecosystems. Bioscience, 52(10), 883. 10.1641/0006-3568(2002)052[0883:ubac]2.0.co;2

[ece310449-bib-0052] McNaughton, S. J. , Oesterheld, M. , Frank, D. A. , & Williams, K. J. (1989). Ecosystem‐level patterns of primary productivity and herbivory in terrestrial habitats. Nature, 341(6238), 142–144. 10.1038/341142a0 2779651

[ece310449-bib-0053] Meineke, E. K. , Classen, A. T. , Sanders, N. J. , & Jonathan Davies, T. (2019). Herbarium specimens reveal increasing herbivory over the past century. Journal of Ecology, 107(1), 105–117. 10.1111/1365-2745.13057

[ece310449-bib-0054] Meineke, E. K. , Dunn, R. R. , & Frank, S. D. (2014). Early pest development and loss of biological control are associated with urban warming. Biology Letters, 10(11), 20140586. 10.1098/rsbl.2014.0586 25411378 PMC4261856

[ece310449-bib-0055] Meineke, E. K. , Dunn, R. R. , Sexton, J. O. , & Frank, S. D. (2013). Urban warming drives insect pest abundance on street trees. PLoS One, 8(3), e59687. 10.1371/journal.pone.0059687 23544087 PMC3609800

[ece310449-bib-0056] Miles, L. S. , Breitbart, S. T. , Wagner, H. H. , & Johnson, M. T. J. (2019). Urbanization shapes the ecology and evolution of plant‐arthropod herbivore interactions. Frontiers in Ecology and Evolution, 7, 310. 10.3389/fevo.2019.00310

[ece310449-bib-0057] Moles, A. T. , Bonser, S. P. , Poore, A. G. B. , & Wallis, I. R. (2011). Assessing the evidence for latitudinal gradients in plant defence and herbivory. Functional Ecology, 25(2), 380–388. 10.1111/j.1365-2435.2010.01814.x

[ece310449-bib-0058] Moles, A. T. , & Westoby, M. (2003). Latitude, seed predation and seed mass. Journal of Biogeography, 30(1), 105–128. 10.1046/j.1365-2699.2003.00781.x

[ece310449-bib-0059] Moreira, X. , Abdala‐Roberts, L. , Berny Mier Y Teran, J. C. , Covelo, F. , de la Mata, R. , Francisco, M. , Hardwick, B. , Pires, R. M. , Roslin, T. , Schigel, D. S. , ten Hoopen, J. P. J. G. , Timmermans, B. G. H. , van Dijk, L. J. A. , Castagneyrol, B. , & Tack, A. J. M. (2019). Impacts of urbanization on insect herbivory and plant defences in oak trees. Oikos, 128(1), 113–123. 10.1111/oik.05497

[ece310449-bib-0060] Moreira, X. , Abdala‐Roberts, L. , Parra‐Tabla, V. , & Mooney, K. A. (2015). Latitudinal variation in herbivory: Influences of climatic drivers, herbivore identity and natural enemies. Oikos, 124(11), 1444–1452. 10.1111/oik.02040

[ece310449-bib-0061] Muiruri, E. W. , Rainio, K. , & Koricheva, J. (2016). Do birds see the forest for the trees? Scale‐dependent effects of tree diversity on avian predation of artificial larvae. Oecologia, 180, 619–630. 26201260 10.1007/s00442-015-3391-6

[ece310449-bib-0062] Nakagawa, S. , & Schielzeth, H. (2013). A general and simple method for obtaining R2 from generalized linear mixed‐effects models. Methods in Ecology and Evolution, 4(2), 133–142. 10.1111/j.2041-210x.2012.00261.x

[ece310449-bib-0063] Noske, R. A. (1998). Breeding biology, demography and success of the rufous‐banded honeyeater, *Conopophila albogularis*, in Darwin, a monsoonal tropical city. Wildlife Research, 25(4), 339–356. 10.1071/wr97070

[ece310449-bib-0064] Novotny, V. , Drozd, P. , Miller, S. E. , Kulfan, M. , Janda, M. , Basset, Y. , & Weiblen, G. D. (2006). Why are there so many species of herbivorous insects in tropical rainforests? Science, 313(5790), 1115–1118. 10.1126/science.1129237 16840659

[ece310449-bib-0065] Nuckols, M. S. , & Connor, E. F. (1995). Do trees in urban or ornamental plantings receive more damage by insects than trees in natural forests? Ecological Entomology, 20(3), 253–260. 10.1111/j.1365-2311.1995.tb00455.x

[ece310449-bib-0066] Oksanen, J. , Simpson, G. , Blanchet, F. , Kindt, R. , Legendre, P. , Minchin, P. , O'Hara, R. , Solymos, P. , Stevens, M. , Szoecs, E. , Wagner, H. , Barbour, M. , Bedward, M. , Bolker, B. , Borcard, D. , Carvalho, G. , Chirico, M. , De Caceres, M. , Durand, S. , … Weedon, J. (2022). vegan: Community ecology package . R package version 2.6‐4. https://CRAN.R‐project.org/package=vegan

[ece310449-bib-0067] Paine, R. T. (1966). Food web complexity and species diversity. The American Naturalist, 100(910), 65–75. 10.1086/282400

[ece310449-bib-0068] Parsons, S. E. , & Frank, S. D. (2019). Urban tree pests and natural enemies respond to habitat at different spatial scales. Journal of Urban Ecology, 5(1), 1–15. 10.1093/jue/juz010

[ece310449-bib-0069] Pebesma, E. , & Bivand, R. (2018). Simple features for R: Standardized support for spatial vector data. The R Journal, 10(1), 439–446. 10.32614/RJ-2018-009

[ece310449-bib-0070] Pennings, S. C. , Ho, C.‐K. , Salgado, C. S. , Wieski, K. , Davé, N. , Kunza, A. E. , & Wason, E. L. (2009). Latitudinal variation in herbivore pressure in Atlantic Coast salt marshes. Ecology, 90(1), 183–195. 10.1890/08-0222.1 19294924

[ece310449-bib-0071] Perez, A. , Chick, L. , Menke, S. , Lessard, J.‐P. , Sanders, N. , Del Toro, I. , Meldgaard, N. S. , & Diamond, S. (2022). Urbanisation dampens the latitude‐diversity cline in ants. Insect Conservation and Diversity, 15(6), 763–771. 10.1111/icad.12598

[ece310449-bib-0072] Price, P. W. (1991). The plant vigor hypothesis and herbivore attack. Oikos, 62(2), 244–251. 10.2307/3545270

[ece310449-bib-0073] R Core Team . (2021). R: A language and environment for statistical computing. R Foundation for Statistical Computing. https://www.R‐project.org/

[ece310449-bib-0074] Remmel, T. , & Tammaru, T. (2009). Size‐dependent predation risk in tree‐feeding insects with different colouration strategies: A field experiment. Journal of Animal Ecology, 78(5), 973–980. 10.1111/j.1365-2656.2009.01566.x 19493131

[ece310449-bib-0075] Rivkin, L. R. , & de Andrade, A. C. (2023). Increased herbivory but not cyanogenesis is associated with urbanization in a tropical wildflower. Austral Ecology, 48(2), 388–398. 10.1111/aec.13274

[ece310449-bib-0076] Romero, G. Q. , Gonçalves‐Souza, T. , Kratina, P. , Marino, N. A. C. , Petry, W. K. , Sobral‐Souza, T. , & Roslin, T. (2018). Global predation pressure redistribution under future climate change. Nature Climate Change, 8(12), 1087–1091. 10.1038/s41558-018-0347-y

[ece310449-bib-0077] Roslin, T. , Hardwick, B. , Novotny, V. , Petry, W. K. , Andrew, N. R. , Asmus, A. , Barrio, I. C. , Basset, Y. , Boesing, A. L. , Bonebrake, T. C. , Cameron, E. K. , Dáttilo, W. , Donoso, D. A. , Drozd, P. , Gray, C. L. , Hik, D. S. , Hill, S. J. , Hopkins, T. , Huang, S. , … Slade, E. M. (2017). Higher predation risk for insect prey at low latitudes and elevations. Science, 356(6339), 742–744. 10.1126/science.aaj1631 28522532

[ece310449-bib-0078] Rueden, C. T. , Schindelin, J. , Hiner, M. C. , DeZonia, B. E. , Walter, A. E. , Arena, E. T. , & Eliceiri, K. W. (2017). ImageJ2: ImageJ for the next generation of scientific image data. BMC Bioinformatics, 18(1), 529. 10.1186/s12859-017-1934-z 29187165 PMC5708080

[ece310449-bib-0079] Salazar, D. , & Marquis, R. J. (2012). Herbivore pressure increases toward the equator. Proceedings of the National Academy of Sciences of the United States of America, 109(31), 12616–12620. 10.1073/pnas.1202907109 22802664 PMC3411992

[ece310449-bib-0080] Sasvári, L. , Csorgo, T. , & Hahn, I. (1995). Bird nest predation and breeding density in primordial and man‐made habitats. Folia Zoologica, 44(4), 305–314.

[ece310449-bib-0081] Schemske, D. W. , Mittelbach, G. G. , Cornell, H. V. , Sobel, J. M. , & Roy, K. (2009). Is there a latitudinal gradient in the importance of biotic interactions? Annual Review of Ecology, Evolution, and Systematics, 40(1), 245–269. 10.1146/annurev.ecolsys.39.110707.173430

[ece310449-bib-0082] Sinclair, R. J. , & Hughes, L. (2010). Leaf miners: The hidden herbivores. Austral Ecology, 35(3), 300–313. 10.1111/j.1442-9993.2009.02039.x

[ece310449-bib-0083] Sirakaya, A. , Cliquet, A. , & Harris, J. (2018). Ecosystem services in cities: Towards the international legal protection of ecosystem services in urban environments. Ecosystem Services, 29, 205–212. 10.1016/j.ecoser.2017.01.001

[ece310449-bib-0084] Sorace, A. (2002). High density of bird and pest species in urban habitats and the role of predator abundance. Ornis Fennica, 79(2), 60–71.

[ece310449-bib-0085] Thorington, K. K. , & Bowman, R. (2003). Predation rate on artificial nests increases with human housing density in suburban habitats. Ecography, 26(2), 188–196. 10.1034/j.1600-0587.2003.03351.x

[ece310449-bib-0086] Tvardikova, K. , & Novotny, V. (2012). Predation on exposed and leaf‐rolling artificial caterpillars in tropical forests of Papua New Guinea. Journal of Tropical Ecology, 28(4), 331–341. 10.1017/S0266467412000235

[ece310449-bib-0087] United Nations . (2018). World Urbanization Prospects 2018 . https://population.un.org/wup/

[ece310449-bib-0088] Valdés‐Correcher, E. , Popova, A. , Galmán, A. , Prinzing, A. , Selikhovkin, A. V. , Howe, A. G. , Mrazova, A. , Dulaurent, A.‐M. , Hampe, A. , Tack, A. J. M. , Bouget, C. , Lupaștean, D. , Harvey, D. , Musolin, D. L. , Lövei, G. L. , Centenaro, G. , Van Halder, I. , Hagge, J. , Dobrosavljević, J. , … Castagneyrol, B. (2022). Herbivory on the pedunculate oak along an urbanization gradient in Europe: Effects of impervious surface, local tree cover, and insect feeding guild. Ecology and Evolution, 12(3), e8709. 10.1002/ece3.8709 35342614 PMC8928871

[ece310449-bib-0089] Whelan, C. J. , Wenny, D. G. , & Marquis, R. J. (2008). Ecosystem services provided by birds. Annals of the New York Academy of Sciences, 1134, 25–60. 10.1196/annals.1439.003 18566089

[ece310449-bib-0090] White, T. C. R. (1969). An index to measure weather‐induced stress of trees associated with outbreaks of psyllids in Australia. Ecology, 50(5), 905–909. 10.2307/1933707

[ece310449-bib-0091] Wickham, H. (2016). ggplot2: Elegant graphics for data analysis (pp. 21–54). Springer‐Verlag. 10.1007/978-0-387-78171-6

[ece310449-bib-0092] Wickham, H. , Averick, M. , Bryan, J. , Chang, W. , McGowan, L. , François, R. , Grolemund, G. , Hayes, A. , Henry, L. , Hester, J. , Kuhn, M. , Pedersen, T. , Miller, E. , Bache, S. , Müller, K. , Ooms, J. , Robinson, D. , Seidel, D. , Spinu, V. , … Yutani, H. (2019). Welcome to the tidyverse. Journal of Open Source Software, 4(43), 1686. 10.21105/joss.01686

[ece310449-bib-0093] Wienert, U. , & Kuttler, W. (2005). The dependence of the urban heat Island intensity on latitude‐a statistical approach. Meteorologische Zeitschrift, 14(5), 677–686.

[ece310449-bib-0094] Youngsteadt, E. , Dale, A. G. , Terando, A. J. , Dunn, R. R. , & Frank, S. D. (2015). Do cities simulate climate change? A comparison of herbivore response to urban and global warming. Global Change Biology, 21(1), 97–105. 10.1111/gcb.12692 25163424

[ece310449-bib-0095] Zvereva, E. L. , Castagneyrol, B. , Cornelissen, T. , Forsman, A. , Hernández‐Agüero, J. A. , Klemola, T. , Paolucci, L. , Polo, V. , Salinas, N. , Theron, K. J. , Xu, G. , Zverev, V. , & Kozlov, M. V. (2019). Opposite latitudinal patterns for bird and arthropod predation revealed in experiments with differently colored artificial prey. Ecology and Evolution, 9(24), 14273–14285. 10.1002/ece3.5862 31938518 PMC6953658

[ece310449-bib-0096] Zvereva, E. L. , & Kozlov, M. V. (2021). Latitudinal gradient in the intensity of biotic interactions in terrestrial ecosystems: Sources of variation and differences from the diversity gradient revealed by meta‐analysis. Ecology Letters, 24(11), 2506–2520. 10.1111/ele.13851 34322961

