# Supplementary material for: Shedding light on trophic interactions: A field experiment on the effect of human population between latitudes on herbivory and predation patterns
Source: Ecol Evol. 2023 Aug 31;13(9):e10449. doi: 10.1002/ece3.10449 (PMC10468994; doi:10.1002/ece3.10449)
Supplement: Supplementary file 1 — Appendix S1. [file ECE3-13-e10449-s001.docx]

Table S1: Coefficients of the best model and standard deviation for bird predation

| Term | β | *SE* | *t* | *p* | 95% CI |
| --- | --- | --- | --- | --- | --- |
| (Intercept) | 1.34 | 0.38 | 3.56 | < .001*** | [0.60, 2.07] |
| HPD | 0.00 | 0.00 | 2.35 | .019* | [0.00, 0.00] |
| RegionSouth | 1.24 | 0.31 | 4.05 | < .001*** | [0.64, 1.85] |
| ColourGreen | -0.62 | 0.24 | -2.60 | .009** | [-1.08, -0.15] |
| ColourYellow | -0.74 | 0.24 | -3.12 | .002** | [-1.20, -0.27] |
| HPD × RegionSouth | -0.00 | 0.00 | -2.09 | .037* | [-0.00, -0.00] |

Table S2: Coefficients of the model-averaged and standard deviation for defoliation herbivory

| Term | β | *SE* | SE2 | *t* | *p* | 95% CI |
| --- | --- | --- | --- | --- | --- | --- |
| cond((Int)) | -3.78 | 0.07 | 0.07 | 54.65 | < .001*** | [-3.92, -3.65] |
| cond(RegionSouth) | 0.20 | 0.08 | 0.08 | 2.48 | .013* | [0.04, 0.36] |
| cond(HPD) | 0.00 | 0.00 | 0.00 | 0.29 | .773 | [-0.00, 0.00] |

Table S3: Coefficients of the model-averaged and standard deviation for miner herbivory

| Term | β | *SE* | SE2 | *t* | *p* | 95% CI |
| --- | --- | --- | --- | --- | --- | --- |
| cond((Int)) | -5.14 | 0.05 | 0.05 | 100.81 | < .001*** | [-5.24, -5.04] |
| cond(RegionSouth) | 0.33 | 0.05 | 0.05 | 6.59 | < .001*** | [0.24, 0.43] |
| cond(HPD) | 0.00 | 0.00 | 0.00 | 0.16 | .872 | [-0.00, 0.00] |

Table S4: Coefficients of the model-averaged and standard deviation for galler herbivory

| Term | β | *SE* | SE2 | *t* | *p* | 95% CI |
| --- | --- | --- | --- | --- | --- | --- |
| cond((Int)) | -3.82 | 0.38 | 0.38 | 10.02 | < .001*** | [-4.57, -3.08] |
| cond(RegionSouth) | 1.89 | 0.44 | 0.44 | 4.34 | < .001*** | [1.04, 2.75] |
| cond(HPD) | 0.00 | 0.00 | 0.00 | 0.58 | .564 | [-0.00, 0.00] |

Table S5: Comparison of alternative models for predation using the Akaike information criterion (AIC) for built surface. The best model (lowest AIC) is indicated in boldface type. R^2^m: a marginal R^2^ that only considers the variability explained by fixed effects; R^2^c: conditional R^2^ that accounts for the variability supported by both fixed and random effects.

|  | Bird predation | | | Insect predation | |
| --- | --- | --- | --- | --- | --- |
| Predictors | *df* | AIC | R^2^m/ R^2^c | *df* | AIC |
| Built * Region * Colour | 14 | 854.26 |  | 13 | 643.48 |
| Built * Region + Colour | **8** | **845.64** | **0.079/0.207** | 7 | 635.05 |
| Built + Region + Colour | 7 | 847.79 |  | 6 | 633.68 |
| Built + Colour | 6 | 858.52 |  | 5 | 632.59 |
| Region + Colour | **6** | **845.79** | **0.072/0.202** | 5 | 631.91 |
| Colour | 5 | 860.06 |  | 4 | 631.75 |
| Null model | 3 | 866.78 |  | **2** | **628.34** |

Table S6: Comparison of alternative models for herbivory using the Akaike information criterion (AIC) for built surface. The best model (lowest AIC) is indicated in boldface type. R^2^m: a marginal R^2^ that only considers the variability explained by fixed effects; R^2^c: conditional R^2^ that accounts for the variability supported by both fixed and random effects.

| Predictors | *df* | AIC | R^2^m/ R^2^c | *df* | AIC | R^2^m/ R^2^c | *df* | AIC | R^2^m/ R^2^c |
| --- | --- | --- | --- | --- | --- | --- | --- | --- | --- |
| Built * Region | 6 | -12,137.15 |  | **6** | **-16,512.12** | **0.022/0.029** | 5 | 2,265.41 |  |
| Built + Region | **5** | **-12,139.07** | **0.008/0.081** | **5** | **-16,513.63** | **0.022/0.029** | **4** | **2,263.45** | **0.156/0.562** |
| Built | 4 | -12,136.76 |  | 4 | -16,476.75 |  | 3 | 2,274.35 |  |
| Region | **4** | **-12,140.92** | **0.008/0.081** | **4** | **-16,512.88** | **0.022/0.029** | **3** | **2,263.16** | **0.145/0.566** |
| Null model | 3 | -12,136.61 |  | 3 | -16,476.86 |  | 2 | 2,281.49 |  |

Table S7: Comparison of alternative models for predation using the Akaike information criterion (AIC) for NDVI. The best model (lowest AIC) is indicated in boldface type. R^2^m: a marginal R^2^ that only considers the variability explained by fixed effects; R^2^c: conditional R^2^ that accounts for the variability supported by both fixed and random effects.

|  | Bird predation | | | Insect predation | |
| --- | --- | --- | --- | --- | --- |
| Predictors | *df* | AIC | R^2^m/ R^2^c | *df* | AIC |
| NDVI * Region * Colour | 14 | 857.51 |  | 13 | 641.30 |
| NDVI * Region + Colour | 8 | 848.22 |  | 7 | 633.57 |
| NDVI + Region + Colour | **7** | **846.57** | **0.072/0.207** | 6 | 633.48 |
| NDVI + Colour | 6 | 861.26 |  | 5 | 633.36 |
| Region + Colour | **6** | **845.79** | **0.072/0.202** | 5 | 631.91 |
| Colour | 5 | 860.06 |  | 4 | 631.75 |
| Null model | 3 | 866.78 |  | **2** | **628.34** |

Table S8: Comparison of alternative models for herbivory using the Akaike information criterion (AIC) for NDVI. The best model (lowest AIC) is indicated in boldface type. R^2^m: a marginal R^2^ that only considers the variability explained by fixed effects; R^2^c: conditional R^2^ that accounts for the variability supported by both fixed and random effects.

| Predictors | *df* | AIC | R^2^m/ R^2^c | *df* | AIC | R^2^m/ R^2^c | *df* | AIC | R^2^m/ R^2^c |
| --- | --- | --- | --- | --- | --- | --- | --- | --- | --- |
| NDVI * Region | **6** | **-12,139.23** | **0.010/0.081** | 6 | -16,509.65 |  | 5 | 2,266.66 |  |
| NDVI + Region | **5** | **-12,140.75** | **0.010/0.081** | 5 | -16,510.88 | **0.021/0.029** | 4 | 2,265.16 | **0.145/0.566** |
| NDVI | 4 | -12,136.86 |  | 4 | -16,475.02 |  | 3 | 2,283.39 |  |
| Region | **4** | **-12,140.92** | **0.008/0.081** | 4 | -16,512.88 | **0.021/0.029** | 3 | 2,263.16 | **0.145/0.566** |
| Null model | 3 | -12,136.61 |  | 3 | -16,476.86 |  | 2 | 2,281.49 |  |

Table S9: Tree species detected in every study site and the total number of individuals per site.

| **Site** | **01** | **02** | **03** | **04** | **05** | **06** | **07** | **08** | **09** | **10** | **11** | **12** | **13** | **14** | **15** | **16** | **17** | **18** |
| --- | --- | --- | --- | --- | --- | --- | --- | --- | --- | --- | --- | --- | --- | --- | --- | --- | --- | --- |
| *Acer platanoides* | 0 | 0 | 52 | 0 | 0 | 0 | 0 | 0 | 0 | 0 | 36 | 0 | 0 | 6 | 0 | 2 | 0 | 0 |
| *Acer pseudoplatanus* | 0 | 6 | 0 | 0 | 0 | 0 | 8 | 0 | 1 | 1 | 0 | 0 | 0 | 0 | 0 | 0 | 0 | 0 |
| *Aesculus hippocastanum* | 0 | 0 | 1 | 0 | 0 | 0 | 0 | 0 | 0 | 0 | 0 | 0 | 0 | 0 | 0 | 0 | 0 | 0 |
| *Betula pendula* | 0 | 0 | 0 | 0 | 0 | 1 | 0 | 0 | 0 | 0 | 0 | 0 | 0 | 0 | 0 | 0 | 0 | 0 |
| *Carpinus betulus* | 5 | 0 | 0 | 0 | 0 | 1 | 0 | 0 | 0 | 13 | 0 | 0 | 0 | 0 | 6 | 12 | 0 | 0 |
| *Crataegus laevigata* | 1 | 0 | 0 | 0 | 0 | 0 | 0 | 0 | 0 | 0 | 0 | 0 | 0 | 0 | 0 | 0 | 0 | 0 |
| *Crataegus monogyna* | 0 | 2 | 0 | 0 | 0 | 0 | 0 | 0 | 0 | 0 | 0 | 0 | 0 | 0 | 2 | 0 | 0 | 0 |
| *Fagus sylvatica* | 3 | 1 | 4 | 8 | 18 | 5 | 12 | 2 | 12 | 13 | 3 | 7 | 6 | 11 | 3 | 4 | 3 | 8 |
| *Frangula alnus* | 0 | 0 | 0 | 0 | 0 | 0 | 0 | 0 | 0 | 0 | 0 | 0 | 0 | 0 | 1 | 0 | 0 | 0 |
| *Fraxinus excelsior* | 0 | 0 | 0 | 0 | 0 | 0 | 0 | 0 | 0 | 0 | 1 | 0 | 0 | 0 | 0 | 1 | 0 | 0 |
| *Lonicera xylosteum* | 0 | 0 | 22 | 0 | 0 | 0 | 0 | 0 | 0 | 0 | 0 | 0 | 0 | 0 | 0 | 0 | 0 | 0 |
| *Pinus sylvestris* | 0 | 1 | 0 | 2 | 0 | 0 | 1 | 0 | 0 | 0 | 0 | 0 | 1 | 2 | 0 | 0 | 0 | 2 |
| *Prunus padus* | 0 | 0 | 0 | 0 | 0 | 0 | 0 | 0 | 0 | 0 | 0 | 1 | 0 | 0 | 6 | 0 | 0 | 0 |
| *Prunus serotina* | 0 | 7 | 0 | 0 | 0 | 0 | 0 | 0 | 0 | 0 | 1 | 1 | 0 | 1 | 0 | 0 | 0 | 2 |
| *Prunus spinosa* | 0 | 0 | 3 | 0 | 0 | 0 | 0 | 0 | 0 | 0 | 0 | 1 | 0 | 0 | 0 | 0 | 0 | 0 |
| *Quercus robur* | 2 | 1 | 0 | 0 | 1 | 0 | 0 | 0 | 0 | 0 | 1 | 0 | 0 | 0 | 1 | 0 | 3 | 1 |
| *Robinia pseudoacacia* | 0 | 0 | 2 | 0 | 0 | 0 | 0 | 0 | 0 | 0 | 0 | 0 | 0 | 0 | 1 | 0 | 0 | 0 |
| *Rosa gymnocarpa* | 0 | 2 | 0 | 0 | 0 | 0 | 0 | 0 | 0 | 0 | 0 | 0 | 0 | 0 | 0 | 0 | 0 | 0 |
| *Rubus ulmifolius* | 0 | 0 | 0 | 0 | 0 | 0 | 0 | 0 | 0 | 0 | 0 | 0 | 1 | 0 | 0 | 0 | 0 | 0 |
| *Sambucus nigra* | 1 | 0 | 0 | 0 | 0 | 0 | 0 | 0 | 0 | 0 | 0 | 0 | 0 | 0 | 0 | 0 | 0 | 0 |
| *Symphoricarpos albus* | 0 | 1 | 0 | 0 | 0 | 0 | 0 | 0 | 0 | 0 | 0 | 0 | 0 | 0 | 0 | 0 | 0 | 0 |
| *Ulmus glabra* | 0 | 2 | 11 | 0 | 1 | 0 | 0 | 0 | 0 | 0 | 1 | 0 | 0 | 0 | 0 | 3 | 0 | 0 |

Figure S1: Biplot showing tree species composition between plots of each region. Predicted values of the species two-dimensional spatial configuration with regard to NMDS1 and NMDS2 axes are shown. Polygons show differential species composition between North and South plots according to the NMDS analysis. Each point represents a study site.

Table S10: Bird species detected in every study site either by sound or directly seen between May and July 2022.

| Site | 01 | 02 | 03 | 04 | 05 | 06 | 07 | 08 | 09 | 10 | 11 | 12 | 13 | 14 | 15 | 16 | 17 | 18 |
| --- | --- | --- | --- | --- | --- | --- | --- | --- | --- | --- | --- | --- | --- | --- | --- | --- | --- | --- |
| *Carduelis carduelis* |  |  |  |  |  |  |  |  | x |  |  |  |  | x |  |  |  |  |
| *Coloeus monedula* |  |  | x |  |  |  |  |  |  |  |  |  |  |  |  |  |  |  |
| *Columba palumbus* |  | x | x |  |  |  | x | x | x | x | x |  |  |  |  | x |  | x |
| *Cyanistes caeruleus* | x |  |  |  |  |  | x |  |  |  |  | x | x | x | x | x | x |  |
| *Dendrocopos major* | x |  |  |  |  |  |  | x |  |  |  |  |  |  |  |  |  |  |
| *Erithacus rubecula* |  |  |  |  |  |  |  |  |  |  |  |  |  |  |  |  |  | x |
| *Fringilla coelebs* | x | x | x | x | x | x | x | x | x | x | x |  |  |  | x | x |  | x |
| *Garrulus glandarius* |  | x |  |  |  |  |  |  |  |  |  |  |  |  |  | x |  |  |
| *Parus major* | x | x |  | x | x | x | x | x |  |  | x |  | x | x |  | x | x |  |
| *Passer domesticus* |  |  |  |  |  |  |  | x |  |  |  |  |  |  |  |  |  |  |
| *Regulus ignicapilla* |  |  | x |  |  |  |  |  |  |  |  |  |  |  |  |  |  |  |
| *Sylvia atricapilla* |  |  |  | x |  | x |  | x |  |  | x |  |  | x |  |  |  |  |
| *Troglodytes troglodytes* |  |  | x |  |  | x |  |  |  |  |  |  |  |  |  |  |  |  |
| *Turdus merula* | x | x | x | x | x | x | x | x | x | x | x | x | x | x | x | x | x |  |

Figure S2: Biplot showing bird species composition between plots of each region. Predicted values of the species two-dimensional spatial configuration with regard to NMDS1 and NMDS2 axes are shown. Polygons show differential species composition between North and South plots according to the NMDS analysis. Each point represents a study site.
